# Supplementary material for: Oestrogen-dependent hypothalamic oxytocin expression with changes in feeding and body weight in female rats
Source: Commun Biol. 2022 Sep 5;5:912. doi: 10.1038/s42003-022-03889-6 (PMC9445083; doi:10.1038/s42003-022-03889-6)
Supplement: Supplementary file 4 — Reporting Summary [file 42003_2022_3889_MOESM4_ESM.pdf]

nature research

Corresponding author(s): Yoichi Ueta

Last updated by author(s): Aug 8, 2022

Reporting Summary

Nature Research wishes to improve the reproducibility of the work that we publish. This form provides structure for consistency and transparency in reporting. For further information on Nature Research policies, see our Editorial Policies and the Editorial Policy Checklist.

Statistics

For all statistical analyses, confirm that the following items are present in the figure legend, table legend, main text, or Methods section.

n/a	Confirmed	
☐	☒	The exact sample size (n) for each experimental group/condition, given as a discrete number and unit of measurement
☐	☒	A statement on whether measurements were taken from distinct samples or whether the same sample was measured repeatedly
☐	☒	The statistical test(s) used AND whether they are one- or two-sided<br>*Only common tests should be described solely by name; describe more complex techniques in the Methods section.*
☒	☐	A description of all covariates tested
☐	☒	A description of any assumptions or corrections, such as tests of normality and adjustment for multiple comparisons
☐	☒	A full description of the statistical parameters including central tendency (e.g. means) or other basic estimates (e.g. regression coefficient) AND variation (e.g. standard deviation) or associated estimates of uncertainty (e.g. confidence intervals)
☒	☐	For null hypothesis testing, the test statistic (e.g. F, t, r) with confidence intervals, effect sizes, degrees of freedom and P value noted<br>*Give P values as exact values whenever suitable.*
☒	☐	For Bayesian analysis, information on the choice of priors and Markov chain Monte Carlo settings
☒	☐	For hierarchical and complex designs, identification of the appropriate level for tests and full reporting of outcomes
☒	☐	Estimates of effect sizes (e.g. Cohen's d, Pearson's r), indicating how they were calculated

Our web collection on statistics for biologists contains articles on many of the points above.

Software and code

Policy information about availability of computer code

Data collection	Fluorescence microscope (ECLIPSE E 600; Nikon Corp., Tokyo, Japan) equipped with an mRFP1 filter (Nikon Corp.)
Data analysis	Imaging analysis system (NIS-Elements; ECLIPSE E 600; Nikon Corp., Tokyo, Japan).

For manuscripts utilizing custom algorithms or software that are central to the research but not yet described in published literature, software must be made available to editors and reviewers. We strongly encourage code deposition in a community repository (e.g. GitHub). See the Nature Research guidelines for submitting code & software for further information.

Data

Policy information about availability of data

All manuscripts must include a data availability statement. This statement should provide the following information, where applicable:
- Accession codes, unique identifiers, or web links for publicly available datasets
- A list of figures that have associated raw data
- A description of any restrictions on data availability

The data that support the findings of this study are available from the corresponding author, upon reasonable request.

Field-specific reporting

Please select the one below that is the best fit for your research. If you are not sure, read the appropriate sections before making your selection.

☒ Life sciences ☐ Behavioural & social sciences ☐ Ecological, evolutionary & environmental sciences

For a reference copy of the document with all sections, see nature.com/documents/nr-reporting-summary-flat.pdf

Life sciences study design

All studies must disclose on these points even when the disclosure is negative.

Sample size	The sample size was chosen on the basis of prior studies that showed significant effects with similar sample sizes.
Data exclusions	Female rats with disturbed estrus cycles were excluded.
Replication	Replicated experiments were successful and support conclusions drawn in this report.
Randomization	Samples were chosen randomly from the OXT-mRFP1 transgenic rat and Wistar rat.
Blinding	Blinding was not possible as experimental conditions were evident from the image data.

Reporting for specific materials, systems and methods

We require information from authors about some types of materials, experimental systems and methods used in many studies. Here, indicate whether each material, system or method listed is relevant to your study. If you are not sure if a list item applies to your research, read the appropriate section before selecting a response.

Materials & experimental systems

n/a	Involved in the study	
☐	☒	Antibodies
☒	☐	Eukaryotic cell lines
☒	☐	Palaeontology and archaeology
☐	☒	Animals and other organisms
☒	☐	Human research participants
☒	☐	Clinical data
☒	☐	Dual use research of concern

Methods

n/a	Involved in the study	
☒	☐	ChIP-seq
☒	☐	Flow cytometry
☒	☐	MRI-based neuroimaging

Antibodies

Antibodies used	Primary antibody solution (goat c-Fos, Santa Cruz Biotechnology, TX, USA; 1:500 or rabbit anti-OXT, Sigma-Aldrich, MO, USA; 1:5000) Secondary antibody (Alexa Fluor 546 donkey anti-goat IgG or Alexa Fluor 488 donkey anti-rabbit IgG; Molecular Probes, OR, USA; 1:2,000 in PBS containing 0.3% Triton X-100)
Validation	Images of Fos+, OXT+, and Fos+/OXT+ double-labelled cells were counted manually by two researchers who were blinded to avoid bias.

Animals and other organisms

Policy information about studies involving animals; ARRIVE guidelines recommended for reporting animal research

Laboratory animals	Adult male and female OXT-mRFP1 Wistar transgenic rats (aged 10-16 weeks and weighing 223–474 g)<br>Adult female Wistar rats (aged 15-16 weeks and weighing 271–337g)
Wild animals	No wild animals were used in this study.
Field-collected samples	No field-collected samples were used in this study.
Ethics oversight	All experiments were performed in strict accordance with guidelines on the use and care of laboratory animals as set forth by the Physiological Society of Japan and approved (No.AE10-012) by the Ethics Committee of Animal Care and Experimentation of University of Occupational and Environmental Health, Japan.

Note that full information on the approval of the study protocol must also be provided in the manuscript.
